# Gene Regulation Shifts Shed Light on Fungal Adaption in Plant Biomass Decomposers

**DOI:** 10.1128/mBio.02176-19

**Published:** 2019-11-19

**Authors:** Jiwei Zhang, Kevin A. T. Silverstein, Jesus David Castaño, Melania Figueroa, Jonathan S. Schilling

**Affiliations:** a Department of Plant and Microbial Biology, University of Minnesota, Saint Paul, Minnesota, USA; b Department of Bioproducts and Biosystems Engineering, University of Minnesota, Saint Paul, Minnesota, USA; c Minnesota Supercomputing Institute, University of Minnesota, Minneapolis, Minnesota, USA; d Department of Plant Pathology, University of Minnesota, Saint Paul, Minnesota, USA; e CSIRO Agriculture and Food, Canberra, Australia; Cornell University

**Keywords:** brown rot adaption, comparative transcriptomics, gene regulation shift, plant biomas, wood-decomposing fungi

## Abstract

Fungi dominate the recycling of carbon sequestered in woody biomass. This process of organic turnover was first evolved among “white rot” fungi that degrade lignin to access carbohydrates and later evolved multiple times toward more efficient strategies to selectively target carbohydrates—“brown rot.” The brown rot adaption was often explained by mechanisms to deploy reactive oxygen species (ROS) to oxidatively attack wood structures. However, its genetic basis remains unclear, especially in the context of gene contractions of conventional carbohydrate-active enzymes (CAZYs) relative to white rot ancestors. Here, we hypothesized that these apparent gains in brown rot efficiency despite gene losses were due, in part, to upregulation of the retained genes. We applied comparative transcriptomics to multiple species of both rot types grown across a wood wafer to create a gradient of progressive decay and to enable tracking temporal gene expression. Dozens of “decay-stage-dependent” ortho-genes were isolated, narrowing a pool of candidate genes with time-dependent regulation unique to brown rot fungi. A broad comparison of the expression timing of CAZY families indicated a temporal regulatory shift of lignocellulose-oxidizing genes toward early stages in brown rot compared to white rot, enabling the segregation of oxidative treatment ahead of hydrolysis. These key brown rot ROS-generating genes with iron ion binding functions were isolated. Moreover, transcription energy was shifted to be invested on the retained GHs in brown rot fungi to strengthen carbohydrate conversion. Collectively, these results support the hypothesis that gene regulation shifts played a pivotal role in brown rot adaptation.

## Abstract (precis)

Fungi dominate the turnover of wood, Earth’s largest pool of aboveground terrestrial carbon. Fungi first evolved this capacity by degrading lignin to access and hydrolyze embedded carbohydrates (white rot). Multiple lineages, however, adapted faster reactive oxygen species (ROS) pretreatments to loosen lignocellulose and selectively extract sugars (brown rot). This brown rot “shortcut” often coincided with losses (>60%) of conventional lignocellulolytic genes, implying that ROS adaptations supplanted conventional pathways. We used comparative transcriptomics to further pursue brown rot adaptations, which illuminated the clear temporal expression shift of ROS genes, as well as the shift toward synthesizing more GHs in brown rot relative to white rot. These imply that gene regulatory shifts, not simply ROS innovations, were key to brown rot fungal evolution. These results not only reveal an important biological shift among these unique fungi, but they may also illuminate a trait that restricts brown rot fungi to certain ecological niches.

## INTRODUCTION

Fungi dominate the turnover of wood, Earth’s largest aboveground terrestrial carbon pool, and they promise commercially relevant pathways for deconstructing lignocellulose. The mechanisms of these fungi vary, with some removing lignin to gain access to cellulose and hemicellulose (white rot type) and others selectively extracting carbohydrates (brown rot type). These nutritional modes represent a spectrum of carbohydrate selectivities (1), but there is a threshold of wood lignin loss that divides white rot (lignin loss is higher) from brown rot (2, 3), and as names imply, color is a distinguishing feature. Furthermore, wood solubilities (3), lignin demethylation patterns (4), and strength loss rates (5) in brown-rotted wood all reflect fungal mechanisms that are distinct from the mechanisms of white rot fungi.

Despite these reliable physiochemical distinctions between brown- and white-rotted wood (phenotypes), it remains unclear what genetic adaptations or mechanism shifts enable these distinct pathways. One obstacle is that brown rot fungi have evolved independently from at least seven different white rot lineages (6, 7). Model fungi from different lineages have been used to inform our working model for brown rot, but often in isolation and with mixed conclusions. Unfortunately, as genomes have become increasingly available, comparative genomics of wood-degrading fungi (8–11) have not identified clear, unified features that distinguish rot types. Another obstacle involves a paradox between lignocellulolytic genes and decay rates. It is evident that the evolution of brown rot fungi involved shedding lignin-degrading peroxidases (PODs) (1, 12, 13) and contracting the number of genes coding carbohydrate-active enzymes (CAZYs), including glycoside hydrolases (GHs) (7, 14). Having fewer genes to encode familiar decay enzymes, however, has not slowed brown rot relative to white rot. Brown rot is typically faster than white rot, and this is exemplified by a standard wood durability test that requires shorter incubation periods for brown rot fungi than for white rot fungi (15).

This contradiction between brown rot fungal gene losses and efficiency gains (the “more-with-less” paradox) could be explained by novel genes or regulatory mechanisms (explanation 1), higher expression of the few genes retained (explanation 2), or some combination of both (explanation 3). Most have suggested novel pathways (explanation 1) are the key to brown rot, specifically mechanisms to deploy reactive oxygen species (ROS) as a pretreatment ahead of CAZYs. This has been posited for decades (16) and repeatedly supported by biochemical evidence (17–19), with iron-dependent Fenton chemistry as a unifying feature (20, 21). This ephemeral (<48 h) ROS pretreatment step has also been isolated, physically (22), to reveal a brief upregulation of lignocellulose oxidation (LOX) genes at the hyphal front of *Postia placenta* (23). These “genes of interest” include some important to white rot mechanisms (e.g., GMC oxidoreductases for H_2_O_2_ production [24]) and some linked to Fenton chemistry (e.g., phenolate-derived iron reductants [25]), relevant for brown rot. The functions of most of these genes, however, are poorly described or unknown and would require significant time/risk to characterize one by one, given a lack of precedent to inform annotations. Gene-by-gene approaches would also inherently assume that novel pathways, specifically ROS-related genes, are the key to brown rot (explanation 1).

Our goal with this study was to harness an experimental setup that could narrow the pool of novel ROS pathway targets (per explanation 1), but that also addressed explanations 2 and 3 as alternate hypotheses. To do this, we grew brown and white rot fungi from distinct clades along the length of wood wafers, and assessed the whole transcriptome from sections removed at set distances behind the hyphal front. This provided a time series of gene expression for tracking differentially expressed genes and for overlaying with wood physiochemical data and enzyme activities. By using these data for functional genomics comparisons among species, we reduced 10-fold the pool of candidate genes unique to brown rot fungal ROS pathways, a major advance in its own right. By looking at the temporal expression, we found the clear shift of LOX genes toward functioning in early decay stages in brown rot compared to white rot. By binning transcripts into functional groups, we also observed a surprising distinction in energy investments between white and brown rot fungi. It was apparent that the brief investment by brown rot fungi in oxidative mechanisms enabled a much higher transcript investment in CAZYs. This supports hypothesis 3, explaining brown rot innovations as a combination of novel pathways and deeper expression investments, and it implies that the evolution of ROS pathways by brown rot fungi did not obsolesce hydrolytic enzymes—it enabled them.

## RESULTS

### Temporal sequence of wood decay among test species.

**FIG 1:**
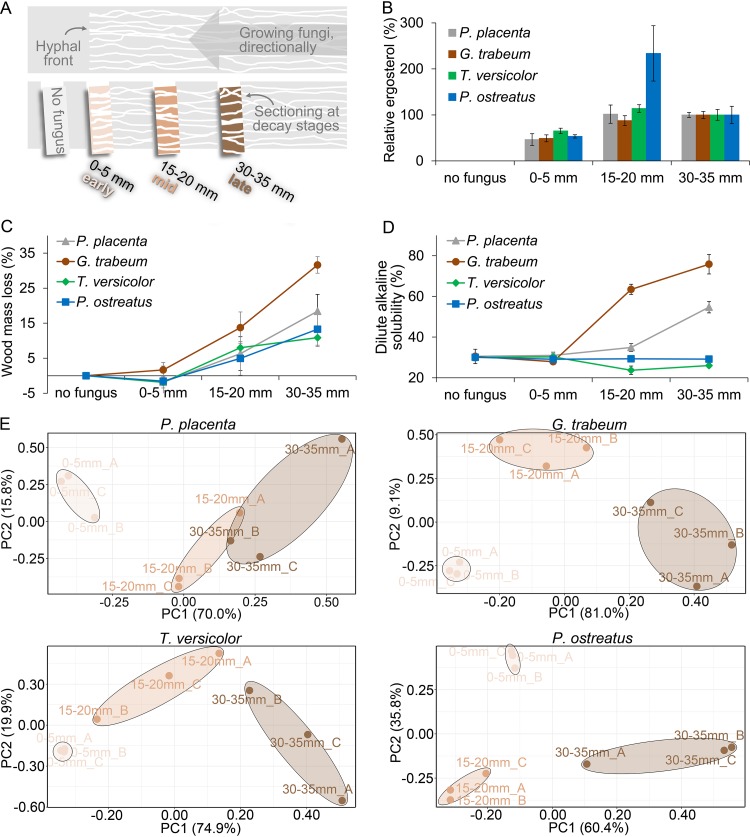
A fine-scale time series of wood decay using directed fungal growth in wafers. (A) Wood decay stages during brown rot (by *P. placenta* and *G. trabeum*) or white rot (by *T. versicolor* and *P. ostreatus*) were manually sectioned in aspen wafers, including a section without fungal growth (no fungus) and three sections behind the hyphal front (0 to 5 mm, 15 to 20 mm, and 30 to 35 mm). A total of 24 independent wafers (*n* = 8, each represented) were sectioned per fungus per experiment, using the hyphal front to benchmark progression. A fungal biomarker, ergosterol (B), is shown relative to late-decay-stage levels (as %) and indicates significant biomass in the 0- to 5-mm “early” section. Wood mass loss (C) and dilute alkaline solubility of wood (D [a sensitive test of early brown rot modifications]) were measured to assess the decay intensity, and neither could distinguish the 0- to 5-mm section from sound wood (ANOVA HSD test, *P* > 0.1). These results, combined, verified that 0- to 5-mm sections were well-colonized but lacked discernible decay, and principal-component analyses (E) clearly distinguished the whole-genome expression patterns in this “early” section (0 to 5 mm) from those in advanced-decay sections (15 to 20 mm and 30 to 35 mm) for all four test fungi. Three bioreplicates from each section (i.e., A, B, and C) were well clustered, indicating the reproducibility of the RNA-seq results.

Four late-diverging fungi (relative to the evolution of other brown rot clades from white rot ancestors) from distant clades were studied, representing brown rot (*Postia placenta* and Gloeophyllum trabeum) and white rot (Trametes versicolor and Pleurotus ostreatus) nutritional modes. All four fungi, when grown directionally, from one end of a wood wafer toward the other end (Fig. 1A), created spatial gradients of decay with uniform hyphal fronts that benchmarked the hyphal front section and enabled reconstruction of time series. These sections included “early decay” at the hyphal front (0 to 5 mm; 30- to 50-h growth period) where fungal biomass (via ergosterol) was abundant but where wood mass losses were negligible or there was apparent mass gain as fungal biomass accrued ahead of wood decay (Fig. 1B and C). The dilute alkaline solubility (DAS) test could not distinguish 0- to 5-mm sections from nondecayed wood for any of the fungi tested, but as decay progressed, DAS increasingly distinguished brown from white rot wood modifications (Fig. 1D).

Transcriptomic data by transcriptome sequencing (RNA-seq) indicated that 77 to 87% of genes in the genomes of the fungi tested were expressed when these fungi decayed solid wood (see Fig. S1 in the supplemental material and Data Set S1 available at https://www.ncbi.nlm.nih.gov/geo/query/acc.cgi?acc=GSE108189), and principal-component analyses (PCA) of transcriptomes clearly revealed the distinct upregulation patterns in early decay zones, relative to older sections for all fungi tested (Fig. 1E). These RNA-seq data were validated with quantitative reverse transcription-PCR (qRT-PCR) using 57 genes relevant for lignocellulose degradation (see Fig. S2 in the supplemental material and Data Set S2 available at https://www.ncbi.nlm.nih.gov/geo/query/acc.cgi?acc=GSE108189) and further used to reannotate genomes (Fig. S1). Collectively, this enabled downstream analyses of differential gene regulation between early and later decay stages, and it will enable others going forward.

10.1128/mBio.02176-19.1 FIG S1 Gene expression evidence and quality control of the newly annotated wood-decaying fungal genomes in this work. (a) Gene expression evidence (RPKM of >5), based on incorporating RNA-seq reads from this wafer design, shown for gene models used in this work. Gene expression found in all three, one or two, or no wood sections is presented as “full,” “partial,” or “none,” respectively. (b and c) Functional annotations by (b) Gene Ontology (Go) and (c) InterPro domain searching for the improved gene models used by this work. (d) By counting the fraction of RNA-seq reads that were accurately mapped to exons, comparison of gene annotation databases revealed improvements in the gene models used in this work relative to preexisting models developed at the Joint Genome Institute (JGI). Download FIG S1, TIF file, 2.2 MB. Copyright © 2019 Zhang et al. 2019 Zhang et al. This content is distributed under the terms of the Creative Commons Attribution 4.0 International license.

10.1128/mBio.02176-19.2 FIG S2 Verification of the RNA-seq results by qRT-PCR in brown rot (*P. placenta* and *G. trabeum* [a and b, respectively]) and white rot (*T. versicolor* and *P. ostreatus* [c and d, respectively]) fungi. The expression levels of 57 lignocellulose-degrading genes were measured by qRT-PCR at sections of 30 to 35 mm and 0 to 5 mm, with three independent bioreplicates. The ratio of qPCR values from these two sections was calculated and used to compare with that of RNA-seq. The correlation coefficient (Cr) was calculated between the data sets from two different mRNA quantitative evaluations, using the “CORREL” function in Microsoft Excel 2016. Download FIG S2, TIF file, 2.8 MB. Copyright © 2019 Zhang et al. 2019 Zhang et al. This content is distributed under the terms of the Creative Commons Attribution 4.0 International license.

### Differentially expressed genes over fungal decay sequences.

**FIG 2:**
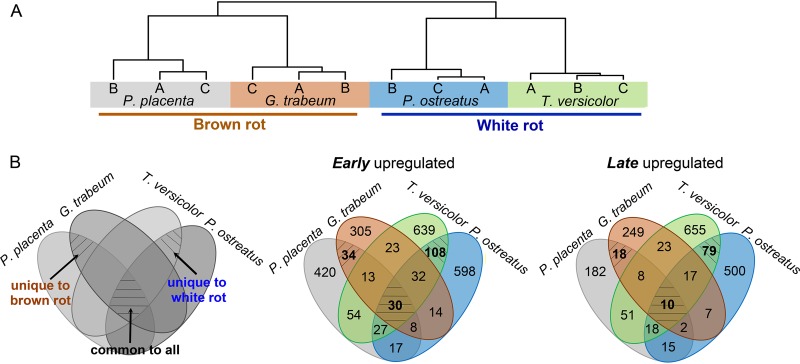
Differential gene expression as a function of species and wood decay stage. (A) The similarity of global temporal gene regulation among species clearly indicated two major clusters distinguished by nutritional modes of brown rot and white rot. The temporal gene expression trends of 2,093 orthologous DEGs were used to calculate the correlations among fungal samples by Spearman’s method. “A,” “B,” and “C” represent triple bioreplicates of RNA-seq data in each species. (B) Unique and common differentially expressed genes (DEGs) as a function of species and wood decay stage. The “decay-stage-dependent” genes, i.e., genes upregulated “early” or “late,” were compared among four species with the orthologous genes. The same MCL ID was assigned to genes from one ortho group, while species-specific ID was used for that without orthologues in other species. “Late” DEGs were combined from 15- to 20-mm and 30- to 35-mm sections. The brown rot fungi are *P. placenta* and *G. trabeum*, and the white rot fungi are *T. versicolor* and *P. ostreatus*.

“Decay-stage-dependent” differentially expressed genes (DEGs) (fold changes [FC] of >4; false-discovery rate [FDR] of <0.05; *n* = 3) in early or late decay stages were assigned for each species (see detailed criteria in Text S1 in the supplemental material), by following the decreasing or increasing expression trends along with decay, respectively (see Fig. S3 in the supplemental material and Data Set S3 available at https://www.ncbi.nlm.nih.gov/geo/query/acc.cgi?acc=GSE108189). The global temporal gene expression trends were immediately compared among species using ortho-DEGs, which display two major clusters more likely distinguished by nutritional modes (i.e., brown rot and white rot), rather than by fungal taxonomy (1, 2, 7, 10) (Fig. 2A).

10.1128/mBio.02176-19.3 FIG S3 Expression patterns of “decay-stage-dependent” DEG subgroups of four tested fungi. The “decay-stage-dependent” DEGs (see Data Set S3 available at https://www.ncbi.nlm.nih.gov/geo/query/acc.cgi?acc=GSE108189) were assigned to eight subgroups according to criteria in Text S1 in the supplemental material. Each gene’s expression, shown as Z-score normalized RPKM values across sections, is plotted in black, with the mean expression profile for each gene subgroup plotted in red. A, section 0 to 5 mm; B, section 15 to 20 mm; C, section 30 to 35 mm. The DEGs with expression trends of groups I, II, III, and IV showed upregulation at the advancing hyphal front (i.e., 0 to 5 mm), and these were combined as early upregulated genes. Comparatively, genes with expression trends of groups V, VI, VII, and VIII were upregulated at later decay sections (i.e., 15 to 20 mm and 30 to 35 mm) and were pooled as late upregulated genes. Download FIG S3, TIF file, 2.1 MB. Copyright © 2019 Zhang et al. 2019 Zhang et al. This content is distributed under the terms of the Creative Commons Attribution 4.0 International license.

10.1128/mBio.02176-19.7 TEXT S1 Supplemental information. Download Text S1, DOCX file, 0.05 MB. Copyright © 2019 Zhang et al. 2019 Zhang et al. This content is distributed under the terms of the Creative Commons Attribution 4.0 International license.

Among these “decay-stage-dependent” genes, those with (hemi)cellulose-degrading *O*-glycosyl hydrolase functions (e.g., GO:0004553 and GO:0008810) were significantly overrepresented in late decay for all four fungi (FDR <0.05, Gene Ontology [GO] enrichment analysis by Fisher’s exact test) (see Table S1 in the supplemental material). Notably, iron ion binding functions (GO:0005506) were significantly overrepresented in early decay for both brown rot fungi, but also enriched in late decay stages for white rot fungi. This indicated the divergences of the temporal gene regulations between two wood-decaying modes besides the similarities.

10.1128/mBio.02176-19.8 TABLE S1 Gene Ontology enrichment analysis of decay-stage-dependent DEGs in wood-decaying fungi. Download Table S1, XLSX file, 0.07 MB. Copyright © 2019 Zhang et al. 2019 Zhang et al. This content is distributed under the terms of the Creative Commons Attribution 4.0 International license.

### Brown rot-specific DEGs.

By using orthologous genes (the ortho group can be found in Data Set S1 available at https://www.ncbi.nlm.nih.gov/geo/query/acc.cgi?acc=GSE108189), we further pinpointed 52 “decay-stage-dependent” DEGs that distinguished brown from white rot species (Fig. 2B; Table 1), which greatly reduced the pool of genes of interest (e.g., those potentially driving ROS production) by more than 10-fold, relative to our previous study (23).

**TABLE 1:** Differentially expressed genes unique to the brown rot fungal strains tested

Gene^*a*^	Functional description^*b*^	*G. trabeum*		*P. placenta*	
		RPKM	FC^*c*^	RPKM	FC^*c*^
Early upregulated					
MCL_5471	Hypothetical membrane protein	427.2	75.9	282.0	12.5
MCL_7627	SAM-dependent methyltransferase	958.5	57.4	44.3	9.2
MCL_3182	Membrane integrating ferric reductase	169.1	16.3	100.3	12.2
MCL_187	bZIP transcription factor	125.2	14.9	95.6	5.9
MCL_7588	Ribonuclease h-related	33.3	11.3	15.0	4.3
MCL_562	Zn(2)-C6 fungal transcription factor	74.3	9.4	60.5	28.3
MCL_1312	Hypothetical membrane protein	2,182.6	8.6	233.5	4.9
MCL_4304	Receptor of activated protein kinase C	554.0	5.7	650.6	5.0
MCL_5286	SRP-dependent cotranslational protein	264.3	5.4	750.2	10.2
MCL_7002	Hypothetical membrane protein SignalP-TM	266.8	5.2	39.4	10.1
MCL_5385	Hyaluronan/mRNA-binding protein	677.0	5.1	357.2	4.8
19 MCLs^*e*^	Ribosomal structure	18,084.9	4.9	16,973.8	4.8
MCL_47	NmrA-like protein	26.2	4.9	60.2	10.2
MCL_1200	bZIP transcription factor	47.8	4.3	19.9	5.7
MCL_2183	Heme-thiolate peroxidase/peroxygenase	90.1	4.3	25.8	4.1^*d*^
MCL_384	Hypothetical protein	186.1	4.1^*d*^	233.3	4.2^*d*^

Late upregulated					
MCL _7542	GH5, endo-β-1,4-glucanase, SignalP-noTM	10,613.2	24.1	12,208.0	10.7
MCL_2441	GH3, β-glucosidase, SignalP-noTM	3,309.1	21.6	2,982.9	5.0
MCL_2166	NAD(P)-binding oxidoreductase	765.7	8.6	963.4^*d*^	5.3^*d*^
MCL_4879	Aldo keto reductase	1,298.5	7.9	945.9^*d*^	5.6^*d*^
MCL_2248	GH3, β-glucosidase	487.1	7.7	639.3	6.5
MCL_5876	Uncharacterized UPF0261	37.2	6.9	138.8^*d*^	9.2^*d*^
MCL_6280	Uncharacterized UPF0303	678.8	6.9	1,871.6	8.7
MCL_2280	Thioredoxin	1,585.8	5.8	3,622.7	5.9
MCL_7782	K(+) channel subunit/aldo keto reductase	4,302.2	5.5	1,197.9	10.6
MCL_3957	Malate synthase, glyoxysomal	1,490.2^*d*^	5.4^*d*^	1,028.4^*d*^	5.1^*d*^
MCL_3499	NADP-dependent 3-OH acid dehydrogenase	1,014.0^*d*^	5.0^*d*^	1,607.1	6.7
MCL_7264	Carboxyl esterase; SignalP-noTM	617.2^*d*^	4.8^*d*^	1,459.4^*d*^	8.0^*d*^
MCL_6643	Sugar transporter	423.6^*d*^	4.7^*d*^	1,065.4	4.1
MCL_1455	Malic enzyme	235.3^*d*^	4.4^*d*^	181.9	4.4
MCL_5447	Peptidyl-prolyl *cis*-*trans* isomerase	3,444.1	4.3	4,415.2	9.0
MCL_2204	Aldo keto reductase	2,030.3	4.3	1,376.7^*d*^	7.9^*d*^
MCL_3421	Aldo keto reductase	1,140.9^*d*^	4.1^*d*^	538.6	5.1
MCL_2530	Glyoxalase/peptidase	130.7^*d*^	4.0^*d*^	599.1	4.5

a Genes are shown by the orthologous group ID generated by OrthoMCL v2.0.

b Functions of orthologues were annotated by Blast2Go (v4.0.7), which combined the results of Gene Ontology annotation and InterPro Scan. Functions were further manually confirmed by blastp in Swiss-Prot database.

c FC, fold change. Ratios were calculated by comparing the RPKM value (reads per kilobase of transcript per million mapped reads) of early decay (i.e., 0- to 5-mm section) and that of the older section (i.e., 15- to 20-mm or 30- to 35-mm section). The gene list was ranked according to the ratio numbers of *G. trabeum*.

d The RPKM value of the 15- to 20-mm section is listed or used to calculate the ratio.

e Nineteen orthologous groups have similar functions for ribosomal structure and were combined to simplify the table.

Thirty-four of these DEGs showed functions unique to early brown rot, coincident with the timing of ROS-based pretreatments. These included the genes encoding ferric reductase (FER; MCL_3182) and heme-thiolate peroxidase/peroxygenase (HTP; MCL_2183) (Table 1; see Fig. S4 and Fig. S5 in the supplemental material). This brown rot FER has a “NOX_Duox_like_FAD_NADP” domain that may catalyze the generation of ROS (e.g., H_2_O_2_) (NCBI Conserved Domains, E value of <3.06e−38), making it plausible that brown rot fungi have coopted the NOX (NADPH oxidase) domain in similar fashion as some pathogenic fungi that infect plants and degrade cellulose (26, 27). The brown rot unique HTP might be involved in halogenating, hydroxylating aromatic molecules and cleaving lignin, as found in *Caldariomyces fumago* chloroperoxidases and *Agrocybe aegerita* aromatic peroxygenases (28–32) (see the Text S1 in the supplemental material for *in silico* functional analysis of FER and HTP). In addition to these two, we also found four “hypothetical proteins,” and four transcriptional factors were uniquely co-upregulated in early brown rot that offer targets to better characterize decaying functions and regulatory mechanisms (Table 1).

10.1128/mBio.02176-19.4 FIG S4 Properties of brown rot unique ferric reductase domain-containing ortholog MCL_3182. (a) Functional Pfam (31.0) domain prediction of two brown rot ferric reductase-containing orthologs (Ppl|g11280.t1 and Gtr|5098.t1) (http://pfam.xfam.org/) that were uniquely upregulated at early decay. (b) Transmembrane domains TM3 to TM5 of NADPH oxidases, ferric reductases, and two brown rot ferric reductase-containing proteins were assigned according to Zhang et al. (70). Conservation logos of NADPH oxidases and ferric reductases were generated by WebLogo 3 (http://weblogo.threeplusone.com/) after amino acid alignment using MafftWS (version 7.310) with preset E-INS-i (accuracy-oriented) and scoring matrix BLOSUM62. The proposed ROS-generating positions (reactive oxygen species) His and Thr-Gly (70), indicated by blue squares, were not present in two brown rot proteins. Download FIG S4, TIF file, 2.2 MB. Copyright © 2019 Zhang et al. 2019 Zhang et al. This content is distributed under the terms of the Creative Commons Attribution 4.0 International license.

10.1128/mBio.02176-19.5 FIG S5 *In silico* analysis of brown rot unique heme-thiolate peroxidase/peroxygenase ortholog MCL_2183. (a)Phylogenetic analysis of fungal heme-thiolate peroxidase/peroxygenase, dye-decoloring peroxidase, and class II peroxidase. Amino acid sequences were aligned using MafftWS (version 7.310) with preset E-INS-i (accuracy oriented) and scoring matrix BLOSUM62 and then used for neighbor-joining tree creation by MEGA7.0.18 with bootstrapping of 1,000. The Poisson correction method was used to compute the evolutionary distances, with less than 5% alignment gaps, missing data, and ambiguous bases at any position. UniProtKB/Swiss-Prot/NCBI accession numbers are shown for the characterized proteins. Class II POD includes VPS (versatile peroxidase), LiP (lignin peroxidase), and MnP (manganese peroxidase). CPO, chloroperoxidase; APO, aromatic peroxygenase; HTP, heme-thiolate peroxidase; Ppl, *P. placenta*; Gtr, *G. trabeum*; Tve, *T. versicolor*; Pos, *P. ostreatus*. Red triangles indicate genes with expression upregulated >4-fold at early decay. Blue triangles indicate genes with expression upregulated >4-fold at late decay. (b) Sequence conservation of brown rot heme-thiolate peroxidase/peroxygenase ortholog MCL_2183. The full length of reference HTP protein sequences was aligned using MafftWS (version 7.310) with preset E-INS-i (accuracy-oriented) and scoring matrix BLOSUM62 and then used to create the conservation logos by WebLogo 3 (http://weblogo.threeplusone.com/). High conservation was highlighted for the proximal heme-binding sequences and heme propionate/cation binding regions. The conserved Cys, indicated by red star, contributes to the heme binding through the thiolate sulfur-iron bond. The predicted α-helix was assigned according to that of *Caldariomyces fumago* CPO (31). Proposed distal acid-base catalyst, acid base pair and Phe sites of substrate binding pocket were assigned per the structures of *Caldariomyces fumago* CPO (31) and *Agrocybe aegerita* APO (32). Download FIG S5, TIF file, 2.5 MB. Copyright © 2019 Zhang et al. 2019 Zhang et al. This content is distributed under the terms of the Creative Commons Attribution 4.0 International license.

Eighteen of these 52 DEGs showed functions unique to late brown rot, upregulated after oxidative modifications of the substrate (17, 22, 23). Specifically, genes for cellulose hydrolysis and methylglyoxal/aldehyde detoxification were upregulated (Table 1), including one endo-β-1,4-glucanase gene (MCL_7542) and two β-glucosidase genes (MCL_2441 and MCL_2248), one carboxyl esterase gene (MCL_7264), and one sugar transporter gene (MCL_6643), along with many aldo keto reductase DEGs. The products of these genes may have evolved characteristics adapted to oxidatively reacted wood components such as carbonyl and carboxylic acid groups introduced on residual carbohydrates (21, 33). In line with this, cellulases and hemicellulases of brown rot fungi were recently shown to better tolerate Fenton-generated oxidative radicals, relative to those enzymes in Trichoderma reesei (34). Overall, these unique late-stage dynamics may involve genes inherited from white rot ancestors (1, 10) with functions adapted to substrate conditions that characterize brown rot, including many unexplored options relevant to industrial processes. In addition, those genes that are “unique to white rot” and “common to all” are discussed in Text S1 in the supplemental material (Fig. 2B; see Table S2 in the supplemental material).

10.1128/mBio.02176-19.9 TABLE S2 Decay-stage-dependent DEGs that are unique to white rot and common in all wood-decaying types. Download Table S2, XLSX file, 0.06 MB. Copyright © 2019 Zhang et al. 2019 Zhang et al. This content is distributed under the terms of the Creative Commons Attribution 4.0 International license.

### Temporal regulation shift of CAZY genes in brown rot versus white rot.

**FIG 3:**
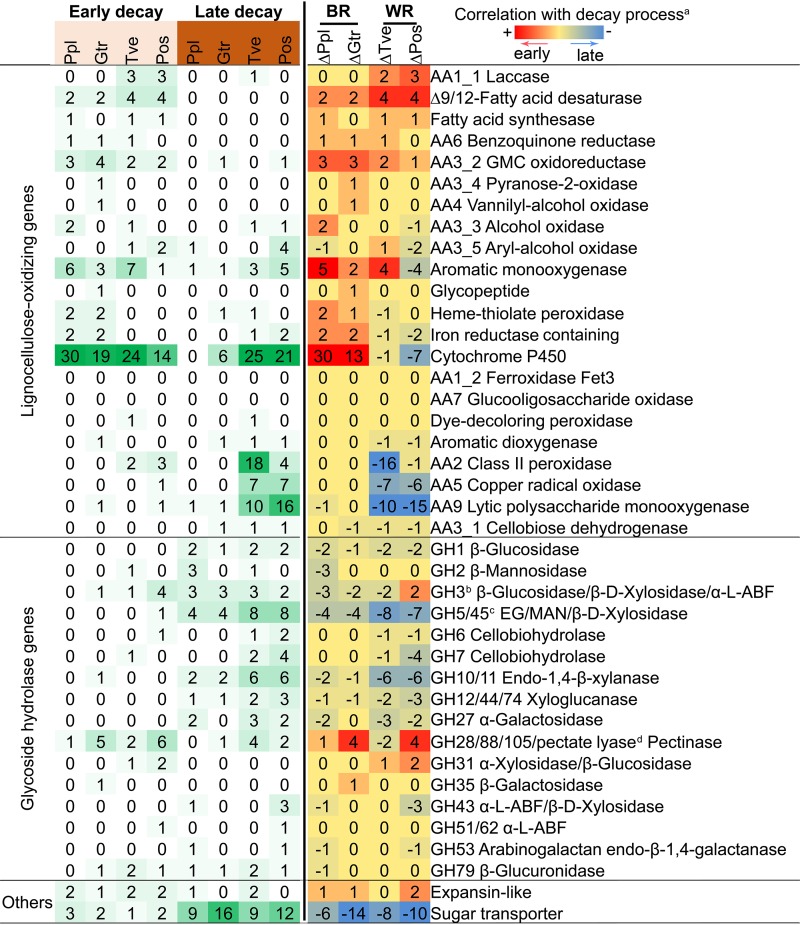
Number of differentially expressed lignocellulose-oxidizing and glycoside hydrolase genes during wood decay by brown or white rot fungi. Genes were manually annotated, primarily according to Carbohydrate-Active enZYmes database (http://www.cazy.org/), verified by blastp with the Swiss-Prot database, and then categorized as lignocellulose-oxidizing or glycoside hydrolase functions. Footnote a indicates correlation of gene expression with decay stage was made by calculating the difference in the number of DEGs between sections with advanced “late” decay (15 to 20 mm plus 30 to 35 mm) and the early decay section (0 to 5 mm). Footnote b indicates *N*-acetyl-β-glucosaminidases were excluded from GH3 family. α-l-ABF, α-l-arabinofuranosidase. Footnote c indicates only EG (endo-β-1,4-glucanase), MAN (endo-β-1,4-mannase), and β-d-xylosidase were included. Footnote d indicates the pectinases in family GH28, -88, and -105 and PL1, -3, -4 were included. Ppl, *P. placenta*; Gtr, *G. trabeum*; Tve, *T. versicolor*; Pos, *P. ostreatus*; BR, brown rot; WR, white rot.

**FIG 4:**
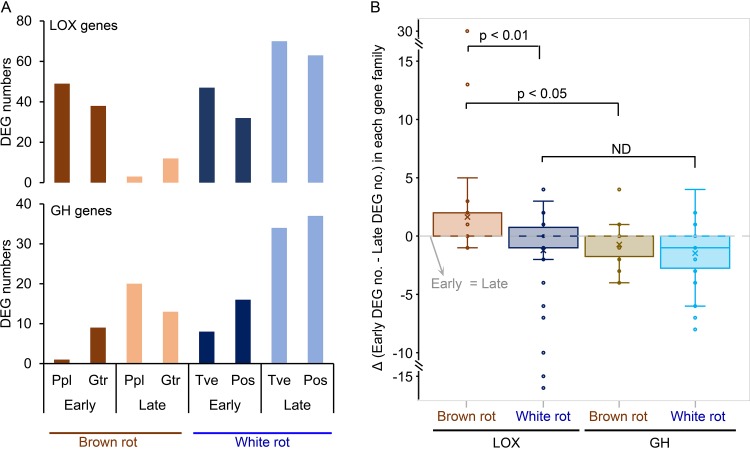
LOX expression shift toward early decay stage in brown versus white rot. (A) Early- or late-upregulated lignocellulose-oxidizing (LOX) and glycoside hydrolase (GH) gene numbers from brown or white rot fungal decay wood. Ppl, *P. placenta*; Gtr, *G. trabeum*; Tve, *T. versicolor*; Pos, *P. ostreatus*. (B) Comparisons of the numbers of early and late DEGs in each CAZY gene family, grouped by wood decay types and functional categories (LOX/GH). Each dot represents the difference value of early and late DEGs in each individual CAZY family. Positive numbers in the *y* axis mean more early DEGs, and the gene functions are more likely associated with early decay, while negative numbers mean the gene functions are tending to be associated with late decay. Significant differences (*P* values) were calculated with a two-tailed paired *t* test. ND, no significant differences were found.

Regulatory patterns of lignocellulose-degrading gene families revealed staggered control of lignocellulose-oxidizing (LOX; upregulated early) and glycoside-hydrolyzing (GH; upregulated later) functions as fungi decayed wood wafers, especially during brown rot (Fig. 3; see Table S3 in the supplemental material). Brown rot LOX genes were, collectively, 10× more upregulated in early than in late decay stages, significantly higher than in white rot, where DEGs were slightly greater in later decay stages (Fisher’s exact test, *P* < 0.001) (Fig. 4A). By further looking at the contrast of early and late DEGs number in each functional CAZY family, it is clear that brown rot LOX expression was shifted to the early decay stage compared to that of white rot (two-tailed paired *t* test, *P* < 0.01 [Fig. 4B]). Specifically, iron-associated genes glycopeptide, heme-thiolate peroxidase, iron reductase, and cytochrome P450 DEGs were more likely correlated with early ROS pretreatment of brown rot, not white rot (Fisher’s exact test, *P* < 0.05) (Fig. 3), in line with the brown rot-specific “decay-stage-dependent” DEGs pinpointed in the Venn diagram (Fig. 2B) and Table 1. The main (hemi)cellulose chain-cleaving GHs (e.g., GH5, -6, -7, -10, and -12) and sugar transporters were generally upregulated during late decay in both brown rot and white rot (early versus late DEGs’ number, two-tailed paired *t* test, *P* < 0.05 [Fig. 4A and B]). These together indicated the distinctive temporal regulatory features in brown rot, which has led the formation of the staggered “two-step” regulation (“LOX-then-GH”) (23).

10.1128/mBio.02176-19.10 TABLE S3 Functional annotation and expression of all CAZY family genes in wood-decaying fungi. Download Table S3, XLSX file, 1.1 MB. Copyright © 2019 Zhang et al. 2019 Zhang et al. This content is distributed under the terms of the Creative Commons Attribution 4.0 International license.

Different from the more defined expression timing of brown rot LOX genes, those in white rot were expressed in both stages of decay. For example, laccases, several aryl-alcohol oxidases, and class II peroxidases were early upregulated only in white rot, which may work for gentler enzymatic pretreatment, in contrast to the harsher ROS pretreatment strategy (e.g., Fenton reaction) in brown rot. On the other hand, many white rot LOX genes, including those encoding 22 class II peroxidases in both white rot species, 26 lytic polysaccharide monooxygenases (LPMO), two cellobiose dehydrogenases (CDH), 14 copper radical oxidases, and two aromatic dioxygenases, were also co-upregulated with GH families in later decay stages (Fig. 3). This coregulation is in line with the simultaneous oxidation of lignin and cellulose at later decay stages during white rot, as well as the saccharification by glycoside hydrolases. The contractions/losses of these genes would likely have shed similar functions among brown rot fungi (2, 7, 9). Again, these distinctive gene expression features reflected the different wood-decaying mechanisms adopted by two classes of fungi (12).

Notable exceptions for GH temporal regulation were found, although they were mainly expressed at late decay stages. For example, several pectin-cleaving enzymes (1 to 6 genes in each species) (35), expansin-like proteins (1 or 2 genes) (36, 37) and some side-chain-cleaving GHs (38, 39) (e.g., GH31 α-xylosidase, GH35 β-galactosidase, GH51 α-l-arabinofuransidase, and GH79 β-glucuronidase) were upregulated early in brown or white rot fungi, suggesting a general requirement among wood decay fungi to loosen plant cell walls in order to facilitate ingress.

### Shift of transcriptional investments toward GH expression in brown rot.

**FIG 5:**
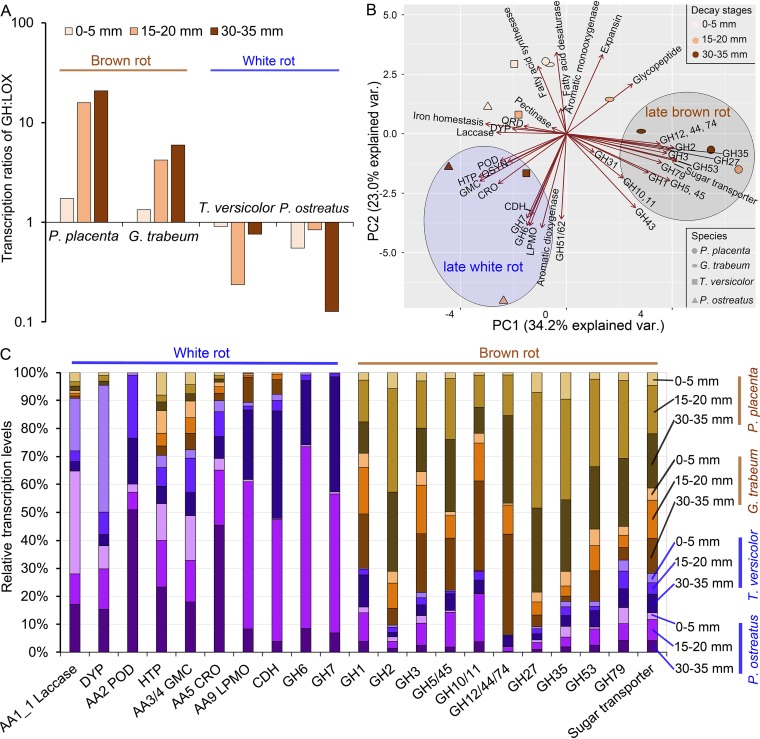
Distinct energy investment strategies of brown versus white rot fungi, reflected by GH transcription levels relative to those of LOX. (A) Transcription ratio of GH to LOX showing brown rot fungi express lignocellulose-degrading GHs at higher levels relative to LOX, particularly as decay progresses. (B) Principal-component analysis of expression levels (RPKM) of lignocellulose-degrading functional gene groups from different decay stages and phenotypes. (C) Relative expression levels of lignocellulose-degrading genes associated with white rot or brown rot, shown as a function of decay stage. QRD, benzoquinone reductase; QSYN, putative hydroquinone synthases, including phenylalanine ammonia-lyase and phenolic 3/4-*O*-methyltransferase; HTP, heme-thiolate peroxidase; DYP, dye-decoloring peroxidase; GMC, glucose-methanol-choline gene family, including AA3_2 GMC oxidoreductase, AA3_3 alcohol oxidase, AA3_4 pyranose oxidase, AA3_5 aryl-alcohol oxidase/pyranose dehydrogenase, AA4_vannilyl-alcohol oxidase, and AA7_glucooligosaccharide oxidase/chitooligosaccharide oxidase. Iron homeostasis includes iron reductase, iron permease, and AA1_2 ferroxidase; POD, class II peroxidase; CRO, copper radical oxidase; LPMO, lytic polysaccharide monooxygenase; CDH, cellobiose dehydrogenase.

Gene expression levels (as reads per kilobase per million [RPKM]) reflected DEG patterns but also revealed a deeper distinction in gene expression investments between brown rot and white rot fungi (Fig. 5; see Data Set S4 available at https://www.ncbi.nlm.nih.gov/geo/query/acc.cgi?acc=GSE108189). In general, the ratio of GH to LOX expression was >1 for brown rot but <1 for white rot irrespective of decay stage, and this ratio was nearly 30-fold higher in brown rot than in white rot in later decay stages (Fig. 5A), indicating brown rot GH expression has been strengthened compared to white rot expression.

Using PCA to analyze gene family expression levels of all decay stages/species further indicated that GH expression tended to be associated with late brown rot, while expression of some LOX and CBH genes was associated with late white rot (Fig. 5B). White rot was characterized by coding gene expansion in families of class II peroxidase, lytic polysaccharide monooxygenase, copper radical oxidase, and laccase (white rot versus brown rot, two-tailed paired *t* test, *P* < 0.005) (Table S3) (1, 7, 9) and had higher expression levels to match gene numbers (Fig. 5C). Brown rot fungi, however, expressed higher levels of GH despite having a contraction of GH-coding genes, such as in families of GH3, GH5/45, GH10/11, GH12/44/74, and GH27 (two-tailed paired *t* test, *P* < 0.001) (Table S3). These RPKM data reveal an energy trade-off that would not be apparent in a genome sequence analysis and that supports hypothesis/explanation 2 (fewer genes retained in genome, but those genes expressed more).

### Extra supports from enzyme presence and specific activities.

**FIG 6:**
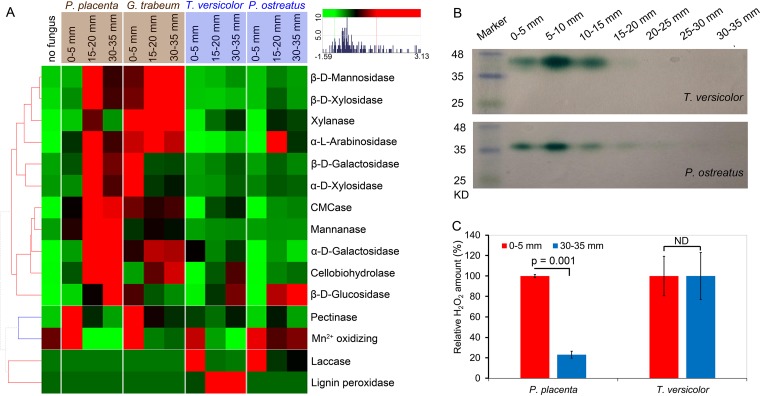
Specific activities of fungal enzymes in wood as a function of decay stage. (A) The specific enzyme activities (IU/μg extracellular proteins [hierarchically clustered]) were measured from sections with no fungus, 0 to 5 mm (i.e., hyphal front), 15 to 20 mm, and 30 to 35 mm, with three bioreplicates pooled from 24 wood wafers (i.e., *n* = 3, each with 8 wafers) (see Text S1 for methods and substrates used for enzymatic assays). The activities were shown as Z-score scaled values: red colors indicate higher levels, while green colors represent lower levels. Hierarchical clustering was made by using the “euclidean” distance matrix and “complete” clustering. (B) The presence and temporal expression of the representative wood-decaying enzyme laccase were confirmed in white rot fungi (*T. versicolor* and *P. ostreatus*) by SDS-PAGE by active staining with 2,2′-azino-bis(3-ethylbenzthiazoline-6-sulfonic acid) (ABTS). (C) Measurements of the wood-oxidizing agent H_2_O_2_ clearly indicated its decreasing trend during brown rot (*P. placenta*), but not white rot (*T. versicolor*). The H_2_O_2_ (nmol/g wood [relative amount]) were measured from sections of 0 to 5 mm and 30 to 35 mm. The significant differences were calculated with two-tailed paired *t* test.

This distinction in GH investment between brown rot and white rot was supported by enzymatic activities, as was the timing (i.e., staggered “two-step”) of the brown rot mechanism. Overall, GH enzyme-specific activities, accounting for the proportion of GH in the total secreted proteins, were clearly higher in brown rot than in white rot (Fig. 6A; see Fig. S6 in the supplemental material). In line with the late upregulation of GH, most of the GH enzymatic activities increased with increasing decay intensity for all fungi, with the exceptions of pectinase and several side chain-cleaving GHs (Fig. 6A; see Fig. S6). As expected, laccase presence (via staining native PAGE gels) was detected only in early white rot (Fig. 6B). The H_2_O_2_ production followed the decreasing trend during brown rot, but not in white rot, in line with the expression patterns of related ROS-producing genes (Fig. 6C).

10.1128/mBio.02176-19.6 FIG S6 Lignocellulose-degrading enzyme activities in wood-decaying fungi. Specific activities of lignocellulose-degrading enzymes, normalized to total secreted proteins (μg), were measured during wood decay with triple bioreplicates pooled from 24 wood wafers (i.e., *n* = 3, each with 8 wafers). The significant differences were calculated with two-tailed paired *t* test. *, *P* < 0.5; **, *P* < 0.01; ***, *P* < 0.001. Download FIG S6, TIF file, 1.7 MB. Copyright © 2019 Zhang et al. 2019 Zhang et al. This content is distributed under the terms of the Creative Commons Attribution 4.0 International license.

## DISCUSSION

Our results narrow the pool of candidate ROS pathway genes by more than 10-fold from earlier work isolating differentially expressed, early upregulated genes, and they confirm in another brown rot fungus *G. trabeum* the same “staggered” LOX-then-GH pattern of gene expression discovered recently in *P. placenta* (23). By comparing with the white rot ancestral expression patterns, this builds a case that the formation of this staggered gene regulatory mechanism in brown rot was to some extent due to the temporal expression shift of LOX genes. This suggests that regulatory rewiring may have contributed to brown rot adaption, including *cis*-regulatory elements and *trans*-acting factors. Studies have shown that the transcription factors have been extensively altered in Basidiomycetes that include most of the wood-decaying fungi, after divergence from Ascomycetes (11).

Differential expression across mycelia colonizing wood likely allows fungi to stage the release of plant cell wall components in a certain order, a sequence that distinguishes brown from white rot deconstruction patterns. A key barrier that both brown and white rot fungi face in degrading plant cell walls is the spatial heterogeneity of the cell wall structure, composed of cellulose microfibrils surrounded by hemicellulose and lignin, as well as pectin and glycoproteins that “gel” the matrix, particularly in the middle lamella (38, 39). To surmount these barriers and release soluble sugars from carbohydrate polymers, brown rot fungi apparently rely on nonenzymatic low-molecular-weight ROS oxidants (e.g.,·OH) to “loosen” the cell wall matrix (40, 41), while white rot fungi confront lignin more directly using ligninolytic peroxidases (42–44). This metabolic distinction reveals itself in the “signatures” we see in wood, where carbohydrate loss outpaces lignin loss during brown rot, and vice versa during white rot (3). Our transcriptomic patterns support this mechanistic distinction between brown and white rot fungi, and our data improve annotations and contribute functional information to enable comparative genomics.

By viewing the whole transcriptome profiles as gene functional clusters, rather as gene-by-gene patterns of differential expression, we found another important and broad distinction between the transcript and enzyme investments of brown and white rot fungi among functional gene classes. Brown rot fungi have been conventionally characterized by contractions in gene families, including GHs (1, 10, 14), yet we observed that the additive expression levels (via RPKM summation) for the small set of GHs in brown rot genomes were consistently and collectively higher than those in the larger set of GHs in white rot fungi (Fig. 5A and C). This likely relates to the fact that white rot fungi must not only invest in more types of GHs but they must also divert energy from GH expression to express PODs, copper radical oxidases (CROs), and LPMOs. This may also explain another distinction between brown and white rot fungi—the loss of carbon catabolite repression (CCR) among brown rot fungi (45). White rot fungi, at least the types we tested here that degrade lignin and carbohydrates at similar rates (simultaneous white rot [3]), may proceed more slowly than brown rot on a strain-by-strain basis due to this requirement for sustained lignin degradation, demanding stricter induction/suppression control of gene expression to save energy. It is logical that a brown rot pathway would offer a fundamental advantage in terms of growth efficiency over a white rot pathway.

Given this advantage, one could argue this might explain why brown rot evolved multiple times from white rot ancestral clades. If, however, brown rot fungi alleviated the need for lignin degradation and gained an energetic advantage over their white rot ancestors, a key question arises: why is white rot more common than brown rot in nature (46)? White rot nutritional modes often dominate fungal communities and wood decay processes in tropical (47) and temperate studies (48), and a meta-analysis of polypore studies (2,428 species records, total) by Ryvarden (49) virtually excluded brown rot from low-elevation tropical and temperate systems below 35°N latitude. This range restriction for brown rot fungi has traditionally been attributed to a substrate preference for conifers (e.g., see reference 49); however, conifers (gymnosperms) are more ancient than angiosperms, while white rot fungi are more ancient than brown rot fungi (6), misaligning coevolution or comigration of brown rot fungi with their conifer tree hosts.

It is possible, instead, that the energetic advantages conferred by the brown rot mechanism are kept in check in nature by ecological, not biological constraints. Community factors, including “cheater” bacteria and fungi that pilfer extricated sugars before absorption by fungi (outlined by Travisano and Velicer [50]), may limit the advantages of a diffuse brown rot pretreatment to environments with less competition such as the higher stress environments found at higher altitudes and latitudes. A streamlining of energy investment in secreted enzymes may only pay off in metabolic “returns” when there is less risk that sugars will be acquired by other organisms before sugars are transported to within the fungal cell wall. Establishing the potential ecological and environmental pressures shaping evolution and limiting the occurrence of brown versus white rot would, in this light, seem an essential pursuit for future research—to link these fungal genomes to the key traits that control this balance of wood rot type on Earth.

Overall, this work implies that brown rot evolution involved shifts in energy investment strategies as much as shifts in gene portfolios or development of novel ROS pathways (supporting explanation 3, outlined in the introduction). This energy return on investment may be a useful framework to understand these fungi in nature, as research in this area moves forward in the fields of biology, ecology, and evolution.

## MATERIALS AND METHODS

### Wood wafer colonization by brown or white rot fungi.

Directional growth of fungi within wood wafers was used to create a sequence of fungal wood decay stages, a space-for-time approach described previously (23). Sterilized aspen (*Populus* sp.) wood wafers (length by width by thickness = 60 by 25 by 2.5 mm) were propped in microcosms with the 25-mm edge resting on the soil surface, colonized from the base by one of four test fungi. The soil medium was a 1:1:1 mixture of soil, peat, and vermiculite hydrated to 40 to 45% (wt/vol) moisture content. Four model wood-decaying Agaricomycetes from distant clades were used in this work, including two brown rot fungi, *Postia* (now *Rhodonia*) *placenta* ATCC 44394 (Polyporales) and *Gloeophyllum trabeum* ATCC 11539 (Gloeophyllales), and two white rot fungi, *Trametes versicolor* A1-ATF (Forest Pathology culture collection, University of Minnesota, USA) (Polyporales) and Pleurotus ostreatus ATCC 32237 (Agaricales). These fungal strains were maintained on 1.5% (wt/vol) malt extract agar prior to inoculating microcosms.

Wafers were harvested when fungi had progressed 50 mm up the length of a wafer. By defining the visible hyphal front as a benchmark (0 mm), four 5-mm sections (5 by 25 by 2.5 mm) were extracted: one from 5 mm above the hyphal front (no fungus), one that included the hyphal front (0 to 5 mm), and two from further behind the hyphal front (15 to 20 and 30 to 35 mm) (Fig. 1A). Three bioreplicates were used by pooling the relevant sections from eight wafers as one bioreplicate. Fungal presence was measured as ergosterol in nondried, fresh wood sections ground in liquid N_2_ (51). Mass loss and dilute alkaline solubility (DAS), indicative of early brown rot wood modifications (22) and distinguishing brown from white rot (3), were determined in oven-dried wood sections (100°C, 48 h), using sawdust (20-mesh) solubility (%) in 0.2 M NaOH (15 min, 121°C) for DAS (3, 22). Significance of differences of the physiochemical results along the advancing mycelium were calculated by one-way analysis of variance (ANOVA) with *post hoc* Tukey’s honestly significant difference (HSD) test (α = 0.05).

### Global gene expression and single-gene validation.

Total fungal RNA was extracted from fresh wood sections with TRIzol (Life Technologies, CA, USA) and cleaned up with a Qiagen RNeasy minikit (Qiagen, Inc., MD, USA) prior to RNA sequencing or quantitative RT-PCR (qRT-PCR), a method validated by Zhang et al. (23). TruSeq RNA v2 libraries (36 in total) were prepared and sequenced on a HiSeq 2500 system (Illumina, Inc., CA, USA) with the standard protocols. Raw RNA-seq reads were trimmed for Truseq2 adapters and for low-quality sequence with Trimmomatic (v0.33) (52), and quality control was integrated using FastQC (v0.11.5) (https://www.bioinformatics.babraham.ac.uk/projects/fastqc/). The remaining reads were mapped to reference genomes using Genomic Short-read Nucleotide Alignment Program (GSNAP) (53). BRAKER1 (v1.8), a recently developed algorithm (54), was used to annotate genomes by incorporating the above-described RNA-seq mapping data. These newly annotated genomes were compared with the Joint Genome Institute (JGI [http://jgi.doe.gov/]) annotations, testing the location of RNA-seq reads relative to exons and significantly improving annotations for all four fungi (Fig. S1). The aligned read counts were computed using the “featureCounts” function of the Rsubread package (v1.22.2) (55) and normalized as reads per kilobase of transcript per million mapped reads (RPKM) for gene expression levels. The test of differential expression was made using edgeR’s (v3.14) “glmQLFit” routine on the design matrix (56). The profiling data for whole-genome transcripts and differentially expressed genes (DEGs) for pairwise comparisons are available in Data Set S1 at https://www.ncbi.nlm.nih.gov/geo/query/acc.cgi?acc=GSE108189 (Gene Expression Omnibus database accession no. GSE108189 [for details, see Text S1 in the supplemental material]).

qRT-PCR was used to verify RNA-seq results by testing expression levels of 57 lignocellulose-degrading genes. Primers were designed with Primer Premier 5.00 (see Data Set S2 available at https://www.ncbi.nlm.nih.gov/geo/query/acc.cgi?acc=GSE108189). The qRT-PCR procedures used in our previous work were applied in this work (23). About 100 ng of total RNA was reverse transcribed to cDNA with a PrimeScript RT reagent kit (Clontech Laboratories, CA, USA) and then used for qPCR with iTaq Universal SYBR green Supermix (Bio-Rad, CA, USA) in an Applied Biosystems 7900HT system (Thermo Fisher Scientific, Inc., MA, USA). Cycle threshold (*C_T_*) values were calculated by Applied Biosystems SDS 2.4.1 software and used for transcription-level estimation by the comparative *C_T_* method, normalizing against β-actin/tef1 genes which have been validated as stably expressed genes (23, 57).

### DEGs in early versus late decay.

With the DEGs obtained by pairwise comparisons among wafer sections, the “decay-stage-dependent” DEGs, i.e., early or late upregulated genes, were defined according to the decreasing or increasing expression trend during decay, respectively (see detailed criteria in Text S1 in the supplemental material). These “decay-stage-dependent” DEGs were compared among fungal species by assigning orthologous groups and overlaying using a Venn diagram (Venny 2.1.0 [http://bioinfogp.cnb.csic.es/tools/venny/]). Orthologous groups among the four fungi were identified via BLAST pairwise reciprocal-best-hit genes and then clustered using OrthoMCL program (v2.0) (58) (see Data Set S1 available at https://www.ncbi.nlm.nih.gov/geo/query/acc.cgi?acc=GSE108189). The orthologous groups for these four tested fungi were well identified, with the majority (62%) containing a single sequence from each of the four fungi. Overall similarities of gene expression trends were compared among species by using the early to late expression ratios of 2,093 ortho-DEGs, which contains all “decay-stage-dependent” DEGs that have orthologues. The early-to-late expression ratios were calculated by log_2_[0- to 5-mm RPKM/avg (15- to 20-mm RPKM, 30- to 35-mm RPKM)] and used to evaluate the correlations among fungal samples by Spearman’s method. Hierarchical clustering of correlation matrix was made by using “euclidean” distance matrix and “complete” clustering in heatmap.2.

### Functional annotation of fungal genomes.

The BRAKER1 annotated protein sequences in this work were queried against the nonredundant (nr) protein sequence database using BLASTP (v2.2.28) (59) (https://blast.ncbi.nlm.nih.gov/), and functional domains were searched with InterProScan (v5.17-56) (60) (http://www.ebi.ac.uk/interpro/). The search outputs of BLASTP and InterProScan were then input into Blast2GO (v4.0.7) (61) to create merged annotation files. More than 70% of genes could be assigned Gene Ontology terms or InterPro domain, indicating the improved genome annotation of this work compared to JGI’s database (Fig. S1).

Focusing on functions related to plant cell wall decomposition, lignocellulose-degrading gene families were manually annotated according to the Carbohydrate-Active enZYmes database (http://www.cazy.org/) and relevant literature (Table S3) and then classified as LOX and GH genes in terms of their oxidative and hydrolyzing roles, respectively. Specifically, these gene families were searched in four tested fungi using blastp (v2.2.28) with well-characterized proteins or by functional domain of the InterPro protein signature database. Gene functions were then verified again via blastp in NCBI using the stringent UniprotKB/Swiss-Prot database, as well as by phylogenetic analyses in Jalview (v2.10.1) (62–64) (see Text S1 in the supplemental material for gene functional annotation). For class II peroxidases (i.e., lignin peroxidase, manganese peroxidase, and versatile peroxidase), the functions were further assigned according to the catalytically active tryptophan and three “Mn binding” acidic amino acid residues (two glutamic acid, one aspartic acid) (7). Expression levels (RPKM) of these gene families were analyzed by principal-component analysis in RStudio (v1.0.143) (http://www.rstudio.org).

### Protein extraction and enzyme activities.

Total extracellular proteins from individual wood sections were extracted in 0.5 M NaCl for 48 h with stirring at 4°C. The extracts were filtered to remove the wood/fungal debris, and protein concentrations were measured using the Bio-Rad protein assay kit (Bio-Rad, CA, USA).

Activities of endoglucanase, xylanase, mannanase, and pectinase were determined as released reducing sugars (via dinitrosalicylic acid reagent) using four model substrates (final concentration of 0.25%): carboxymethyl cellulose, birchwood xylan, locust bean gum (glucomannan), and polygalacturonic acid (65). Lignocellulose-oxidizing enzymes laccase and lignin peroxidase, along with manganese-oxidizing activities, were measured by oxidizing 1 mM 2,2′-azino-bis(3-ethylbenzothiazoline-6-sulfonic acid), 0.03 M azure B, or 1 mM 2,6-dimethoxyphenol, respectively. Cellobiohydrolase, β-d-glucosidase, β-d-xylosidase, α-d-xylosidase, β-mannosidase, β-d-galactosidase, α-d-galactosidase, and α-l-arabinosidase activities were measured by using the corresponding 4-nitrophenyl derivatives as the substrates (66–68). All activities were presented as units per microgram of extracellular proteins (specific activity). The presence of laccase secreted during wood decay was also assessed by native PAGE gel staining with 0.5 mM ABTS [2,2′-azinobis(3-ethylbenzthiazolinesulfonic acid)] as the substrate (66) (see the Text S1 in the supplemental material for more details). Extracellular H_2_O_2_ was measured by using Amplex Red 10 (69).
