# Supplementary material for: Gene Regulation Shifts Shed Light on Fungal Adaption in Plant Biomass Decomposers
Source: mBio. 2019 Nov 19;10(6):e02176-19. doi: 10.1128/mBio.02176-19 (PMC6867892; doi:10.1128/mBio.02176-19)
Supplement: TEXT S1 [file mBio.02176-19-s0001.docx]

**Supplementary information**

***Supplementary Information Narrative***

Contents, by page number (*sub-sections not included*):

**Page Section**

1 1. RNA-seq analyses

2 2. Comparison of stage-dependent genes among species

4 3. Protein function annotation

4 4. In silico analysis of brown rot unique ortholog

6 5. Lignocellulose-degrading enzymes determination

1. ***RNA-seq analyses***

TruSeq RNA v2 barcoded libraries (36 in total, i.e., 4 species × 3 wood sections × 3 bioreplicates; average insertions ≈ 200 bp) were prepared and sequenced on a 125 bp paired-end run on the HiSeq 2500 System (Illumina, Inc.) using v4 chemistry (23). RNA-seq was performed at the University of Minnesota Genomics Center (http://genomics.umn.edu/). A total of ≥260 million reads were generated for each species in a single sequencing flow cell lane, and this generated 20-30 million pass filter reads for each sample.

The RNA-seq reads were analyzed using the following pipeline: 1) reads were cleaned up with Trimmomatic, 2) reads were aligned with Genomic Short-read Nucleotide Alignment Program (GSNAP), 3) genomes were annotated using BRAKER1 by incorporating RNA-seq data, 4) gene expression levels (RPKM) were calculated and differentially-expressed genes (DEGs) were determined using edgeR.

Specific to step one of the analytical pipeline, the raw reads were first trimmed for Truseq2 adapters and for low-quality sequence with Trmmomatic (v0.33) (`ILLUMINACLIP:TruSeq2-PE.fa:2:30:10:8:no LEADING:3 TRAILING:3 SLIDINGWINDOW:4:15 MINLEN:36`) (52). Quality control of the trimmed reads was performed with FastQC (v0.11.5) (https://www.bioinformatics.babraham.ac.uk/projects/fastqc/).

Second, all remaining paired end reads were mapped to the respective reference genome using GSNAP with parameters optimized for somewhat distant sequence identity (`--max-mismatches=40 --gmap-min-match-length=3 --novelsplicing=1 --localsplicedist=5000 --distant-splice-penalty=2`) (53). The reference genomes, including *Postia placenta* MAD-698-R-SB12 v1.0, *Gloeophyllum trabeum* v1.0, *Trametes versicolor* v1.0 and *Pleurotus ostreatus* PC15 v2.0 were downloaded from JGI (Joint Genome Institute, http://jgi.doe.gov/) and used for alignment. The mapping rates reached 99%, 99%, 96% and 88% for *P. placenta*, *G. trabeum*, *T. versicolor* and *P. ostreatus*, respectively.

Third, RNA-seq reads mapping data were used as input to BRAKER1 (v1.8) (54), an improved gene prediction model that combines the strengths of two leading prediction tools, Augustus and GeneMark. BRAKER1 uses GeneMarks ability to train on raw RNA-seq data mappings to the genome in order to learn organism-specific gene characteristics (e.g., intron and exon size distributions, start and stop signals, splice signals). The initial gene models produced by GenMark are then automatically fed into Augustus to improve its base gene discovery. We used SAMtools to sort and then merge all 9 RNA-seq mappings from each organism into a single BAM file. Then BRAKER1 was run using the –fungus option on this merged input file. BRAKER1 produced protein fasta and gene coordinate (gtf) files for each organism (Gene Expression Omnibus database accession no. GSE108189). These newly annotated gene models were compared with JGI’s genome annotation by testing the location of RNA-seq reads relative to exons (**Fig. S1d**).

Fourth, differentially-expressed genes among the three wood sections (0-5 mm, 15-20 mm, 30-35 mm) for each organism were computed using the edgeR package v3.14 (56). Read counts were computed using the `featureCounts` routine (55) in the Rsubread package v1.22.2 (`useMetaFeatures=TRUE, isPairedEnd=TRUE`). To avoid retaining spurious genes in the noise across all samples, genes had to have at least one count-per-million of 5 in 3 of the 9 samples to be tested for differential expression. Reads Per Kilobase of transcript per Million mapped reads (RPKM) was used to represent gene expression level. The test of differential expression was made using edgeR's `glmQLFit` routine with F-test on the design matrix (56). Using the appropriate coefficient from the test, we obtained a ranked list of the most differentially-expressed genes across any pair of conditions (Gene Expression Omnibus database accession no. GSE108189; **Dataset S1**).

1. ***Comparison of stage-dependent genes among species***

***2.1 Definition of ‘decay-stage-dependent’ genes*** The ‘decay-stage-dependent genes’, i.e., early- or late-upregulated genes, were defined according to the decreasing or increasing expression trend during decay, respectively. 1) The differentially-expressed genes for paired wood sections were first selected using a fold change threshold > 4 and FDR < 0.05. 2) These DEGs were then assigned to four decreasing (e.g., І, П, Ш and ІV) and four increasing (e.g., V, VI, VП, VШ) expression trends (GSE108189 **Dataset S3 and Fig. S3**) according to the criteria: **І**, 0-5 mm or 15-20 mm vs. 30-35 mm > 4-fold, FDR < 0.05 and FDR for 15-20 mm vs. 0-5 mm > 0.05; **П**, 0-5 mm vs. 30-35 mm > 4-fold, FDR < 0.05 and 0-5 mm > 15-20 mm, FDR < 0.05 and 15-20 mm > 30-35 mm, FDR < 0.05; **Ш**, 0-5 mm vs. 15-20 mm or 30-35 mm > 4-fold, FDR < 0.05 and 0-5 mm > 15-20 mm, FDR < 0.05 and 0-5 mm > 30-35 mm, FDR < 0.05 and FDR for 30-35mm vs. 15-20 mm > 0.05; **ІV**, 0-5 mm vs. 15-20 mm > 4-fold, FDR < 0.05 and 0-5 mm > 30-35 mm > 15-20 mm, FDR for 30-35 mm vs. 0-5 mm < 0.05; **V**, 30-35 mm vs. 0-5 mm or 15-20 mm > 4-fold, FDR < 0.05 and FDR for 15-20 mm vs. 0-5 mm > 0.05; **VI**, 30-35 mm vs. 0-5 mm > 4-fold, FDR < 0.05 and 15-20 mm > 0-5 mm, FDR < 0.05 and 30-35 mm >15-20 mm, FDR < 0.05; **VП**, 30-35 mm or 15-20 mm vs. 0-5 mm > 4-fold and FDR for 15-20 mm vs 0-5 mm < 0.05 and FDR for 30-35 mm vs 0-5 mm < 0.05 and FDR for 30-35 mm vs 15-20 mm > 0.05; **VШ**, 15-20 mm vs. 0-5 mm > 4-fold, FDR < 0.05 and 30-35 mm < 15-20 mm, FDR < 0.05, or 15-20 mm vs. 0-5 mm > 4-fold, FDR < 0.05 and FDR for 30-35 mm vs. 15-20 mm > 0.05 and FDR for 30-35 mm vs. 0-5 mm > 0.05. Only DEGs in these eight expression trends were analyzed downstream as decay-stage-dependent genes. The DEGs in group I, II, III, IV were combined as early upregulated genes, showing upregulation at the advancing hyphal front (i.e., 0-5 mm). Comparatively, these in group V, VI, VII, VIII were pooled as late upregulated genes, showing upregulated expression at later decay sections (i.e., 15-20 mm and 30-35 mm).

***2.2 Orthologous gene group identification*** To make the comparison of DEGs among different organisms, the orthologous gene groups of four tested fungi were identified. Following the OrthoMCL program (v2.0) (58), a protein blast database was created by prefixing each protein sequence ID with a 3-letter species code, and concatenating all four proteomes into a single fasta file. Then all-vs-all `blastp` (`-evalue 1e-5 -outfmt 7`) was run with the formatted database. A custom perl script was used to identify reciprocal best hits in the tab-delim output file, and suppress the output of all secondary hits. Then clustering of these hits was performed using the fuzzy-logic clustering MCL program (`-I 2.0`) to generate tight orthologous groups. Inspection of these groups revealed that the majority of them (62%) contained a single sequence from each of the 4 fungi, but some had variations (e.g., multiple sequences from one or more fungi added, or missing representatives from one or more fungi) (GSE108189 **Dataset** **S1**). When comparing genes among species, the same MCL ID was assigned to genes from one orthogroup, while species-specific ID was used for that without orthologues in other species.

***2.3 DEGs that are ‘unique to white rot’ and ‘common to all’*** The decay-stage-dependent ortho gene comparisons also revealed 187 DEGs with patterns unique to white rot, and 40 genes with a shared differential regulation pattern among all of the test wood-decaying types.

Among the DEGs in common among the fungi, 30 genes were upregulated in early decay, including 9 for cell proliferation and lipid metabolism. Ten of these shared DEGs were upregulated in later decay stages, including GH12 xyloglucan-specific endo-β-1,4-glucanase (MCL_5209), GH79 β-glucuronidase (MCL_0585) and two sugar transporters (MCL_4895 and MCL_1336) (**Table S2a, b**).

Among the 187 white rot-specific DEGs, 108 were upregulated in early decay (**Table S2c**). Most of these (73 of 108) were associated with basic biological processes such as amino acid/lipid/nucleotide metabolism, protein synthesis, respiration, cell proliferation and cell wall/membrane synthesis. Six genes were also associated with toxin production or detoxification. Among the remaining DEGs upregulated in later decay stages during white rot, 22 of 79 could be linked to lignocellulose-degrading roles, of which eight encode GHs, and eight are auxiliary activities (AA) family genes. This included five AA_9 lytic polysaccharide monooxygenases (**Table S2d**). Unlike the glycosyl hydrolases in late brown rot, 10 of these white rot genes upregulated in later decay stages had cellulose-binding domains (CBDs).

1. ***Protein function annotation***

***3.1 Protein function annotation with Blast2GO*** The protein fasta files of four fungi generated by BRAKER1 (v1.8) were then processed with `blastp v2.2.28` against `nr` (`-outfmt 5 -evalue 1e-3 -word_size 3 -show_gis -num_alignments 20 -msx_hsps_per_subject 20`) (<https://blast.ncbi.nlm.nih.gov/>) (59), and with `interproscan v5.17-56` (<http://www.ebi.ac.uk/interpro/>) (60). The XML output files from these tasks were fed into Blast2GO v3.3 (61), to create merged annotation files. The percentage of gene models with Gene Ontology or InterPro annotations in this work were summarized (**Fig. S1a, b, c**).

***3.2 Functional annotation of lignocellulose-degrading genes*** The lignocellulose-degrading genes, categorized according to lignocellulose-oxidizing (LOX) and glycoside hydrolyzing (GH) functions, were searched among wood-decaying fungi using the following pipeline: 1) BlastP (v2.2.28) search in relevant fungal genomes with the amino acid sequences of well-characterized proteins (**Table S3**), and select the hit sequences with score > 100, 2) search the target InterPro domains (InterPro 56.0) (https://www.ebi.ac.uk/interpro/), 3) search the target genes assigned by Blast2Go (v4.0.7), 4) combine the sequences obtained from step 1, 2 and 3, and 5) verify the sequences from step 4 using BlastP in NCBI (https://blast.ncbi.nlm.nih.gov/Blast.cgi) using the UniprotKB/Swiss-Prot database. Gene names were then assigned according to the following criteria: accept the gene name of database if BlastP score > 200; record as “putative gene” if 200 > score > 80; record as “hypothetical gene” if 80 > score > 50; or record as “possible gene” if score < 50. The final step in this process was phylogenetic analysis in Jalview (v2.10.1) (62) to lend additional verification to gene function (**Table S3**).

Expression levels (RPKMs) of CAZY gene families were analyzed by principle component analysis in RStudio (v1.0.143) (http://www.rstudio.org). Package ‘ggfortify’ (https://cran.r-project.org/web/packages/ggfortify/index.html) was used, and package ‘ggbiplot’ (https://www.rdocumentation.org/packages/ggbiplot/) was used to plot the figure. The raw expression data can be found in “GSE108189_Dataset_S4.Expression_levels_of_lignocellulose-oxidizing_and_glycoside_hydrolase_gene_families.xlsx”. Default parameters used for PCA in R, as ‘prcomp(dataxxx, center = TRUE, scale. = TRUE)’.

1. ***In silico analysis of brown rot unique ortholog***

***4.1 Amino acid alignment***, ***phylogenetic tree and sequence*** ***conservation analysis*** Amino acid sequences were aligned using MafftWS (version 7.310) (63) with preset E-INS-i (accuracy-oriented) and scoring matrix BLOSUM62. Neighbor-Joining trees were created by MEGA7.0.18 (64) with Poisson correction and with bootstrapping of 1000. The sequence conservation logos were created by WebLogo 3 (http://weblogo.threeplusone.com/).

***4.2 In silico analysis of brown rot ferric reductase*** ***ortholog MCL_3182*** InterProScan (5.25-64.0) and Pfam (31.0) searching indicated that *MCL_3182* (*Ppl|11280.t1* and *Gtr*|*5098.t1*) encodes a membrane integrating ferric reductase superfamily protein. This superfamily includes two homologous family enzymes ferric reductases (FRE) and NADPH oxidases (NOX), and both contain a heme-containing 6 transmembrane ferric reductase domain and two C-terminal cytoplasmic FAD-binding and NADPH-binding domains (**Fig. S4a**) (70). FREs catalyze the reduction of ferric iron to ferrous form, while NOXs transfer electrons to oxygen to generate ROS, and NOX enzymes have been proven involved in pathogenicity as fungi infects plant and degrade plant cellulose (26, 27). This brown rot MCL_3182 showed ~25% aa identity to characterized ferric reductase and NADPH oxidase (BlastP with SwissProt database), indicating its possible roles in either transferring electrons to ferric iron or oxygen to generate ROS.

To further identify the function of brown rot MCL_3182, amino acid sequences of 11 characterized FREs and eight NOXs were aligned and the sequence conservation of these two families were analyzed (**Fig. S4b**). The following FERs and NOXs were used: *Botrytis cinerea* NOX1_CAP12516.1, *Botrytis cinerea* NOX2_CAP12517.1, *Podospora anserine* NOX1_CDP22559.1, *Podospora anserina* NOX2_CDP30078.1, *Aspergillus nidulans* NOX1_Q8J0N4, *Epichloe festucae* NOX1_Q2PEP0, *Magnaporthe grisea* NOX1_ABS01490.1, *Magnaporthe grisea* NOX2_ABS01491.1, *Saccharomyces cerevisiae* FRE1_P32791.1, *Saccharomyces cerevisiae* FRE2_P36033.1, *Saccharomyces cerevisiae* FRE3_Q08905.1, *Saccharomyces cerevisiae* FRE4_P53746.1, *Saccharomyces cerevisiae* FRE5_Q08908.1, *Saccharomyces cerevisiae* FRE6_Q12473.1, *Saccharomyces cerevisiae* FRE7_Q12333.2, *Saccharomyces cerevisiae* FRE8_Q12209.1, *Eremothecium gossypii* FRE8_Q75CQ8.1, *Schizosaccharomyces pombe* FRE1_Q04800.1, *Schizosaccharomyces pombe* FRE2_O94727.1. Four His positions and one Phe, contributing to heme binding, were highly conserved in both FERs and NOXs, and exist in brown rot MCL_3182 (Ppl|11280.t1 and Gtr|5098.t1). Although the conserved ROS-generating relevant positions (His and Thr-Gly) (70) were replaced by Arg and Val-Gly in Ppl|11280.t1, and by Lys and Val-Gly in Gtr|5098.t1, the similar ammonium cation of these positively charged amino acids might retain the functions of NOX for ROS generation.

***4.3 In silico analysis of brown rot heme-thiolate peroxidase/peroxygenase*** ***ortholog MCL_2183*** InterProScan (5.25-64.0) and Pfam (31.0) searches showed that MCL_2183 (Ppl|4414.t1 and Gtr|2796.t1) has the peroxidase family 2 domain, and BlastP search in swissprot found it has 30-35% aa identities with *Caldariomyces fumago* CPO_P04963.3 and *Agrocybe aegerita* APO_B9W4V6.1, indicating *MCL_2183* encodes the heme-thiolate peroxidase/peroxygenase. Phylogenetic relationships (**Fig. S5a**) of wood-decaying heme-thiolate peroxidase/peroxygenase (HTP) and class II peroxidase (POD, e.g., LiP, MnP and VP), dye-decoloring peroxidase (DYP) were analyzed by Neighbor-Joining method in MEGA7.0.18 with bootstrapping of 1000, after amino acid alignment using MafftWS (version 7.310) with preset E-INS-i (accuracy-oriented). The following sequences were used: **HTP,** *Caldariomyces fumago* CPO_P04963.3, *Agaricus bisporus* CPO_AJ293759.1, *Agrocybe aegerita* APO_B9W4V6.1, *Coprinellus radians* APO_B9W4V8.2, *Marasmius rotula* APO_5FUJ_A, *Exidia glandulosa* HTP_KZV96010.1, *Pleurotus ostreatus* HTP_KDQ32971.1, *Mycena galopus* HTP _jgi|684634|; **POD,** *Phanerochaete chrysosporiu* MnP1_AAA33742.1, *Phanerochaete sordida* MnP2_BAC06186.1, *Gelatoporia subvermispora* MnP_AAD45725.1, *Gelatoporia subvermispora* MnP_EMD32808.1, *Bjerkandera adusta* VPS_AAY89586.1, *Pleurotus pulmonarius* VPS_AFM93767.1, *Pleurotus eryngii* VPS_3FM6_A, *Phanerochaete chrysosporium* LiP_CAA38177.1, *Trametes versicolor* LiP_AAA34049.1, *Phlebia radiata* LiP_AAW66483.1; **DYP,** *Mycetinis scorodonius* DYP_B0BK71.1, *Mycetinis scorodonius* DYP_B0BK72.1. The HTPs are more distant to the well-known lignin-degrading PODs relative to DYPs, and they are basically categorized into two groups, of which one group clustered with the characterized *Caldariomyces fumago* CPO_P04963.3 and *Agrocybe aegerita* APO_B9W4V6.1(31, 32) and another group contains the early-upregulated MCL_2183 (Ppl|4414.t1 and Gtr|2796.t1).

The conservation logos of HTP proteins were created after aligning the amino acid sequences of the above known HTPs using MafftWS (version 7.310) (**Fig. S5b**). The relevant functional positions were designated according to the structural studies of *Caldariomyces fumago* CPO_P04963.3 and *Agrocybe aegerita* APO_B9W4V6.1 (31, 32). The heme-thiolate peroxidase/peroxygenase (HTP) function of this brown rot MCL_2183 was validated by the typical cysteine as the fifth ligand of the iron in the heme ring (31, 32). Comparing to the characterized HTPs, this brown rot HTP has several substitutions at the Phe position by nonphenolic side-chain amino acids in the proposed substrate-binding pocket, as well as the replacement of distal acid-base catalyst Glu by Asn (**Fig. S5b**), indicating its broader substrate accessibility and changes in specific catalyzing mechanisms, which may be worth further functional study.

***5. Lignocellulose-degrading enzymes determination***

***5.1 Enzyme activity assays*** Lignocellulose-degrading enzyme activities, including oxidoreductases and glycoside hydrolases, were measured for the extracellular proteins to support the gene expression patterns. Crude enzymes (0.5 ml) were tested for endoglucanase, xylanase, mannanase and pectinase activities in 0.1 M acetate buffer (0.5 ml) at pH 4.8 and 50°C, with 0.75% (0.5 ml, final con. 0.25%) of carboxymethyl cellulose, birchwood xylan, locust bean gum (glucomannan) and polygalacturonic acid as substrates, respectively. Reactions were stopped by adding 2 mL of a dinitrosalicylic acid developing reagent (65) and boiled before detection of reducing sugars. Reducing sugars were determined by measuring the absorbance at 540 nm on an iMark microplate reader (Bio-Rad), and calculated by using glucose, xylose, mannose or galacturonic acid standard curves, respectively, depending on the substrate. One unit of activity was defined as the amount of enzyme needed to liberate 1 μmol of sugar equivalent per minute.

The cellobiohydrolase, β-D-glucosidase, β-mannosidase, β-D-xylosidase, α-L-arabinosidase, β-D-galactosidase, α-D-xylosidase or α-D-galactosidase activities were measured for 50 µl of enzyme extract in 0.1 M acetate buffer (50 ul) at pH 4.8 and 50°C, with 50 ul of 2 mg⋅mL^−1^ 4-Nitrophenyl β-D-cellobioside + 330 ug⋅mL^−1^ castanospermine, 3 mg⋅mL^−1^ 4-Nitrophenyl β-D-glucopyranoside, 3 mg⋅mL^−1^ 4-Nitrophenyl β-D-mannopyranoside, 1 mg⋅mL^−1^ 4-Nitrophenyl β-D-xylopyranoside, 1 mg⋅mL^−1^ 4-Nitrophenyl α-L-arabinofuranoside, 3 mg⋅mL^−1^ 4-Nitrophenyl β-D-galactopyranoside, 1 mg⋅mL^−1^ 4-Nitrophenyl α-D-xylopyranoside or 3 mg⋅mL^−1^ 4-Nitrophenyl α-D-galactopyranoside as respective substrates. The 10% Na_2_CO_3_ (150 ul) was added to stop reaction and develop color. The 4-Nitrophenyl derivative substrates were replaced by 0.1 M acetate buffer and used as blank reaction. The released 4-Nitrophenol (pNP) was determined by measuring the absorbance at 420 nm and calculated with pNP standard curve. One unit of activity was defined as the amount of enzyme needed to liberate 1 μg of pNP per minute (23). For *G. trabeum*, α-D-xylosidase, β-D-galactosidase and β-D-glucosidase activities were very high in early brown rot decay, consistent with expression levels of the corresponding gene families.

Laccase activities were determined by measuring the changes of OD_420_ in reactions that contained 40 µl 5mM ABTS, 80 µl citric buffer (pH 3.0) and 80 µl properly diluted crude enzyme. The reactions were run at room temperature for 30 min (for white rot) or 24 h (for brown rot). One unit of laccase activity was defined as the amount of enzyme needed to oxidize one µg ABTS per minute, using the molar extinction coefficient 36000 M^-1^cm^-1^ (66). Only marginal ABTS activities were detected for two brown rot fungi in this work, which is different to our previous observation may due to the variable in wood substrates (23).

The lignin peroxidase activity was measured according to Archibald (1992) (67). The reaction mixture contained 250 µl of 100 mM citric buffer (pH 6.5, optimum pH), 150 µl of 0.16 mM Azure B (BeanTown Chemical Corp.), 100 µl of 1 mM H_2_O_2_, and 300 µl of enzyme extract. The reaction was initiated by adding H_2_O_2_ at room temperature, and the mixture was then incubated for 5 min. The oxidation rate of Azure B was measured with a UV-VIS spectrophotometer (UV-1800) at 613 nm. One unit of activity was defined as the amount of enzyme needed to cause one ∆OD_613_ per minute. Spectrum scan of the absorption of azure B revealed that maximum absorption was at about 613 nm, rather than the 650 nm (67). Lignin peroxidase activities (via Azure B oxidation) were only detected in white rot fungus *T. versicolor*, and were significantly higher in late decay. No obvious lignin peroxidase activities were found in *G. trabeum*, *P. placenta* or *P. ostreatus*.

The manganese (2+) oxidizing ability was measured for crude extract (68) in a reaction mixture containing 600 µl citrate-phosphate buffer (pH 6.5), 50 µl 20 mM MnSO_4_, 50 µl 20 mM 2,6-dimethoxyphenol (DMP), 100 µl enzyme extract, and 200 µl 2 mM H_2_O_2_. The reaction was initiated by adding H_2_O_2_, and the mixture was then incubated at 35°C for 25 min. The absorbance was recorded at 450 nm, and absorbance of mixtures without MnSO_4_ or enzymes were used as controls. One unit of activity was defined as the amount of enzyme needed to cause one ∆OD_450_ per minute. Mn^2+^ oxidization was also detectable without adding enzyme extract, perhaps due to oxidation by H_2_O_2_, which was normalized to extracellular protein amount (µg) of 0-5 mm section for comparison. Accordingly, the activity here reflects the overall manganese (2+) oxidizing ability of crude extract, which might be the combination of positive effects of Mn^2+^ oxidizing agent (e.g., H_2_O_2_) and manganese peroxidase, and negative effects of H_2_O_2_-degrading catalase and fungal anti-oxidizing agents. This Mn^2+^ oxidizing capacity showed a uniform decrease during brown rot, and in the white rot fungus *P. ostreatus*, activity remained high in late decay.

***5.2 Native polyacrylamide gel staining*** The presence of laccase was also tested on native polyacrylamide gel after staining with ABTS solution. Specifically, equal amounts of each crude protein extract (without denaturing) from different decay stages (i.e., wafer sections) were separated by SDS-PAGE (Polyacrylamide gel electrophoresis) using a 5% (wt/vol) polyacrylamide stacking gel and a 12% (wt/vol) polyacrylamide resolving gel. The gel was then washed in 0.05 M acetate buffer (pH 4.2) for 1 h for three times to remove SDS. Laccase was observed by staining the gel in 0.1 g⋅L^−1^ guaiacol or 0.5 mM ABTS in 0.05 M acetate buffer (pH 4.2) for 1.5 h at room temperature (66).
